# Narrow ovale foramina may be involved in the development of primary trigeminal neuralgia

**DOI:** 10.3389/fneur.2022.1013216

**Published:** 2022-10-11

**Authors:** Shuo Li, Chenlong Liao, Meiqiong Qian, Xiaosheng Yang, Wenchuan Zhang

**Affiliations:** ^1^Department of Neurosurgery, Shanghai Ninth People's Hospital, Affiliated to Shanghai Jiao Tong University School of Medicine, Shanghai, China; ^2^Department of Imaging, Shanghai Ninth People's Hospital, Affiliated to Shanghai Jiao Tong University School of Medicine, Shanghai, China

**Keywords:** foramen ovale, primary trigeminal neuralgia, cause, recurrence, microvascular decompression

## Abstract

**Background:**

The etiology of primary trigeminal neuralgia remains unclear and is worthy of further study; In this study, the morphometric characteristics of ovale foramina between various groups were compared and analyzed to explore the novel cause of primary trigeminal neuralgia.

**Methods:**

High-resolution three-dimensional reconstruction images from head computed tomography of 109 patients with primary trigeminal neuralgia affecting the third branch of the trigeminal nerve and 46 healthy controls were retrospectively reviewed. Among the 109 primary trigeminal neuralgia patients, 79 patients with apparent neurovascular compression (not simply contact) demonstrated on MRI or during surgery were divided into the classical trigeminal neuralgia group and 30 patients with MRI showing no significant abnormalities were divided into idiopathic trigeminal neuralgia group. The morphometric parameters including the area, width and length of ovale foramina were examined through the use of radiologic methods.

**Results:**

In this study, the average minimum area, width and length of 79 ovale foramina on the affected and unaffected sides in the classical trigeminal neuralgia group were 21.83 ± 8.45, 21.94 ± 7.93 mm^2^, 2.32 ± 0.91, 2.58 ± 0.81, 5.32 ± 1.29, and 5.26 ± 1.21 mm, respectively. No significant difference in these parameters was observed (*p* > 0.05). However, in the idiopathic trigeminal neuralgia group, the average minimum area, width and length of 30 ovale foramina were 21.33 ± 8.21, 22.85 ± 8.36 mm^2^, 2.25 ± 0.90, 2.79 ± 0.96, 5.20 ± 1.27, and 5.28 ± 1.19 mm, respectively. The width on the symptomatic side was significantly smaller (*p* = 0.03) than that on the asymptomatic side. No significant difference in area (*p* = 0.48) or length (*p* = 0.79) was observed. In addition, when compared with the healthy control group, the area and width of ovale foramina on the symptomatic side in both groups were significantly smaller. No significant difference in length was observed.

**Conclusions:**

By comparing and analyzing the statistical data, it can be inferred that a narrow foramen ovale is associated with primary trigeminal neuralgia, as well as its recurrence after microvascular decompression.

## Introduction

As a functional disorder of the peripheral nervous system, trigeminal neuralgia (TN) is characterized by drastic and electric shock-like orofacial neuropathic pain in the distribution area of the trigeminal nerve (1). TN tends to occur in the elderly, but is also observed in children (2, 3). In 2018, the International Headache Society classified TN into several categories, including classical TN (CTN), idiopathic TN (ITN), and secondary TN (STN) (4). The first two, also termed primary TN (PTN), are distinguished by the degree of neurovascular contact (NVC) (5). NVC has been widely accepted to be the main cause of PTN due to the extremely high rate of postoperative pain relief with microvascular decompression (MVD) (5–9). Nevertheless, there are still some clinical observations and symptoms that fail to be explained by NVC. For example, some PTN patients without NVC have been observed during posterior fossa explorations (10–13), whereas a number of individuals with NVC never present specific clinical manifestations similar to PTN (14–16). Therefore, some researchers have proposed that NVC is not the only etiology of PTN (17–19), which warrants further study and attention.

Anatomically, the foramen ovale is an important aperture in the skull base and transmits the third division of the trigeminal nerve (i.e., the mandibular nerve) and several small vessels (20). Many investigations have reported that foramen ovale has great clinical significance in some manners (21–23). In this study, we aimed to emphasize that the mandibular branch could be entrapped during its course through a narrow foramen ovale, thus suggesting that a narrow foramen ovale may play a role in the development of PTN (20, 24, 25). This perspective was first proposed by Neto et al. (19). In addition, they proposed the idea that a narrow foramen ovale may account for the higher prevalence of PTN on the right side because many anatomical and radiological studies have suggested that the right-sided ovale foramina were narrower than left-sided ovale foramina in most people (26–28).

Clinically, PTN patients with no pain relief or with recurrence after MVD have always represented an intractable medical problem that has not been effectively addressed. Although the high pain relief rate (80–98%) after MVD has been previously reported (29), the postoperative long-term recurrence rate can reach as high as 15–35% (30, 31). Currently, in the case of PTN patients with recurrence, it is still controversial as to whether to perform MVD again or to treat the problem with percutaneous procedures (32–34). In this study, we measured and compared the size of ovale foramina between various groups to explore whether a narrow foramen ovale is associated with the pathogenic mechanism and relapse of PTN, thus helping to provide new insights into the existing surgical procedures.

## Methods and materials

### Data source

In this retrospective study, we screened consecutive TN patients attending the Department of Neurosurgery, Shanghai Ninth People's Hospital, affiliated with Shanghai Jiao Tong University School of Medicine, from January 2019 to January 2022. The diagnosis was based on the Third Edition of the International Classification of Headache Disorders. The inclusion criterion included PTN involving the mandibular branch. The exclusion criteria included STN, skull base fractures, and other headache disorders (such as migraines). A total of 250 TN patients were screened and 141 patients were excluded: 17 with STN, 21 with anatomic variants like bony spur and tubercle bony plate of ovale foramina, and 103 patients with PTN not affecting the mandibular branch. At length, 109 PTN patients consisting of 79 CTN patients and 30 ITN patients were eligible and divided into the CTN group and ITN group according to the degree of NVC which was determined by MRI/MRTA and intraoperative exploration findings. For the sake of the following discussion, the two groups were also referred to as the PTN group. Additionally, we also recruited 46 healthy controls (HCs) as the HC group. The demographics and patient characteristics are listed in Table 1.

**Table 1:** Demographic and pain characteristics of HC, CTN and ITN groups.

	**CTN**	**ITN**	**HC**	**Significance**
**Demographic**
Case number (N/%)	79 (51.0)	30 (19.4)	46 (29.7)	
FO number (N/%)	79 (39.3)	30 (14.9)	92 (45.8)	
**Gender**
Female (N/%)	21 (70.0)	48 (60.8)	29 (63.0)	ns
Male (N/%)	9 (30.0)	31 (39.2)	17 (37.0)	
Age	60.4	65.5	58.2	ns
**Pain characteristics**
**Side**
Right (N/%)	14 (46.7)	42 (53.2)		ns
Left (N/%)	16 (53.3)	37 (46.8)		
**Branch**
V_3_	9 (30.0)	29 (36.7)		
V_2+3_	21 (70.0)	42 (53.2)		
V_1+2+3_	0 (0.0)	8 (10.1)		

ns, no significant difference.

### Imaging technique

Measurements were conducted on high-resolution, thin-slice head computed tomography (CT) images which were obtained by a 256-slice CT (Phillip, Netherlands) scanner and transferred to the Picture Archiving and Communicating System of the Radiology Department. A 3D reconstruction was then created on a post-processing workstation (Advantage Workstation 4.6, GE Healthcare, Milwaukee, WI). After the 3D-CT image of the cranium was obtained, the both-sided ovale foramina can be observed by looking at the cranium from the bottom, and the parameters of each foramen ovale were measured with a self-contained measuring tool.

### Statistical analysis

**Figure 1:**
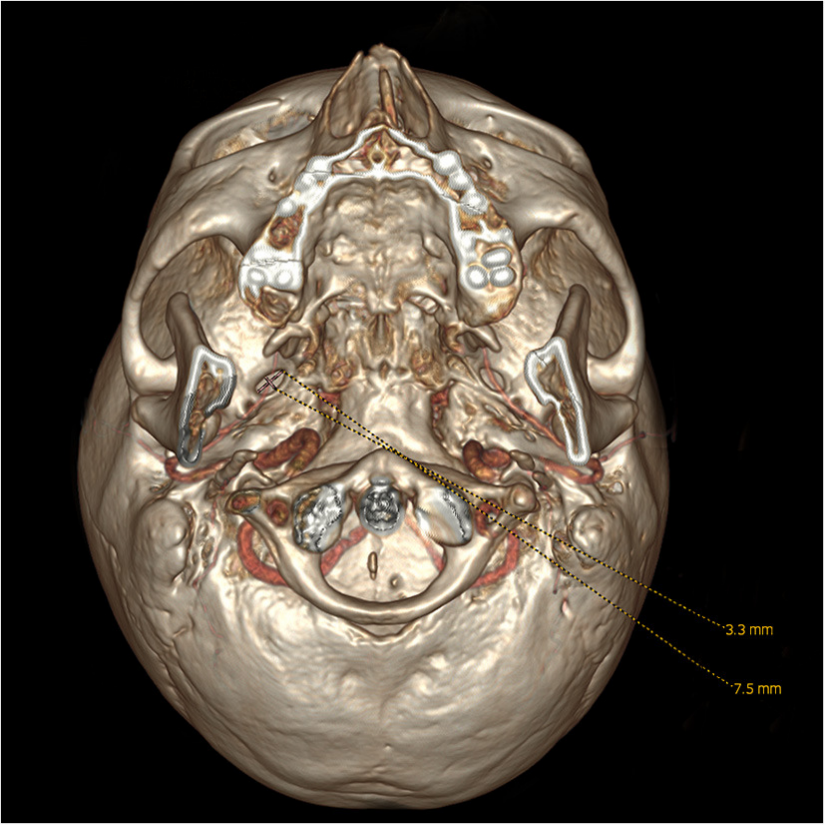
Observation and measurement of the length and width of the foramen ovale from the bottom on 3D reconstruction images.

**Figure 2:**
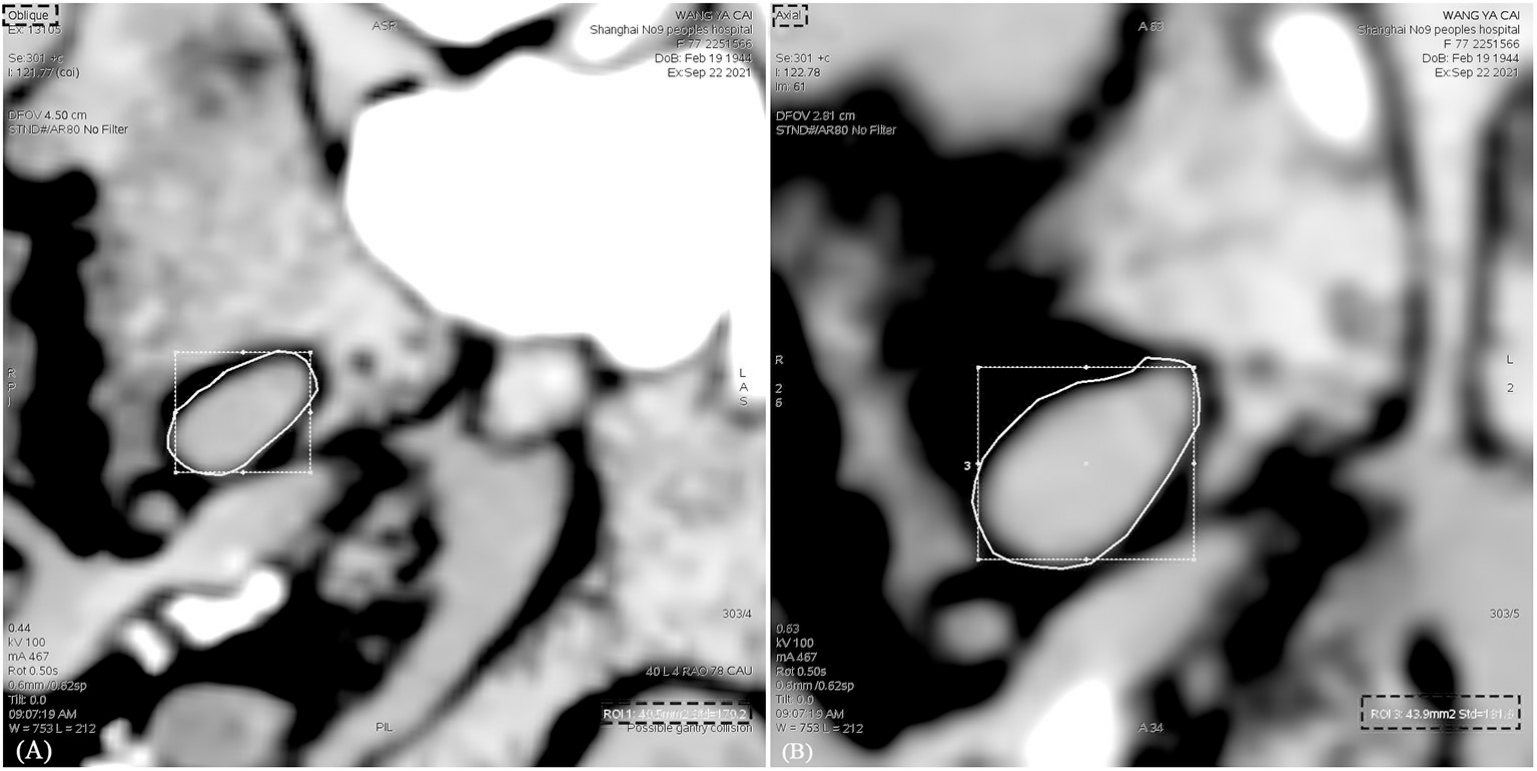
Measurement of the area of the foramen ovale on CT images in different position. **(A)** The area of the foramen ovale was measured in the oblique position. **(B)** The area of the foramen ovale was measured in the axial position. The area measured in the axial position is greater than that in the oblique position.

To avoid a measurement bias, the collection of clinical data and the measurements of ovale foramina were performed by independent neuroradiologists. In this case, the length was defined as the diameter along the longest dimension of each shape, and the width was defined as the perpendicular bisector of length. The length and width of ovale foramina were measured on 3D reconstruction images (Figure 1). To ensure the accuracy of the data and the level that we measured was the smallest level, the area was measured in the oblique position, instead of the axial position, with the angle adjusted to be perpendicular to the outer opening of the foramen ovale (Figure 2). Data were expressed as the mean ± standard deviation (SD) and compared by using one-way analysis of variance (one-way ANOVA) for parameters with normal distributions. A multivariate analysis was then performed by using logistic regression. In addition, constituent ratios of sex and side were examined *via* the chi-square test or Fisher's exact probability test. The abovementioned statistical analyses were performed by using IBM SPSS Version 26.0 (IBM Corp., Armonk, New York, USA). A *p*-value of < 0.05 was considered to be statistically significant.

## Results

### Differences in the morphometry between the left and right sides

The morphometric parameters of ovale foramina in the HC, CTN, and ITN groups were recorded and compared separately between the left side and the right side. The detailed statistical data and corresponding *p*-values are listed in Table 2. Although the mean size of the left-sided ovale foramina was slightly smaller than that of the right-sided ovale foramina in most individuals, there was no significant difference in morphometric characteristics found between the left and right sides. The results suggested that the size of the bilateral ovale foramina was symmetric in either PTN patients or healthy individuals.

**Table 2:** Comparisons of parameters between the left and right sides in HC, CTN and ITN groups.

**Parameter**	**Group**	**Left**	**Right**	***P*-value**
Area (mm^2^)	HC	24.90	27.28	0.19
	CTN	21.47	22.30	0.52
	ITN	23.21	21.60	0.44
Width (mm)	HC	2.64	2.77	0.39
	CTN	2.45	2.45	0.99
	ITN	2.49	2.55	0.81
Length (mm)	HC	5.27	5.79	0.04^*^
	CTN	5.22	5.36	0.46
	ITN	5.10	5.38	0.79

* *p* < 0.05.

### Differences in the morphometry between the painful and painless sides

**Figure 3:**
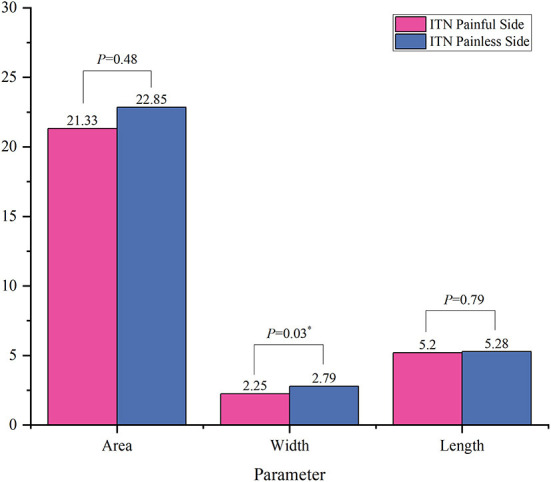
Morphometric differences between the painful side and the painless side in the ITN group. *p < 0.05.

**Figure 4:**
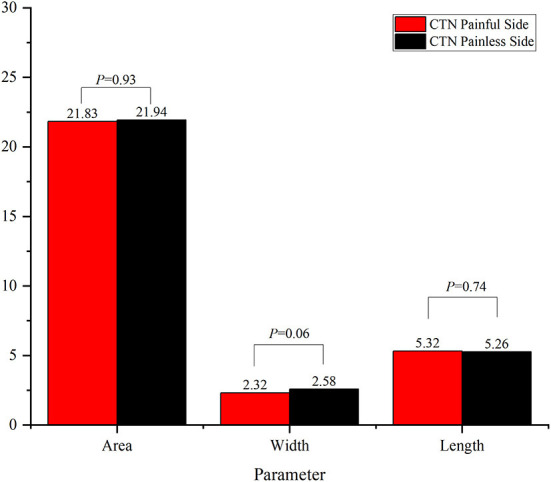
Morphometric differences between the painful side and the painless side in the CTN group.

The results of comparison of foramen ovale size ipsilateral and contralateral to the side of symptoms are shown in Figures 3, 4. In the ITN group, the width of ovale foramina on the painful side was significantly narrower than that of the painless-sided ovale foramina (*p* = 0.03), whereas no significant difference in the area and length was observed. Distinct from the results of the ITN group, the symptomatic and asymptomatic sides in the CTN group did not present any statistically significant differences in all morphometric parameters.

### Comparison of parameters between the three groups

**Figure 5:**
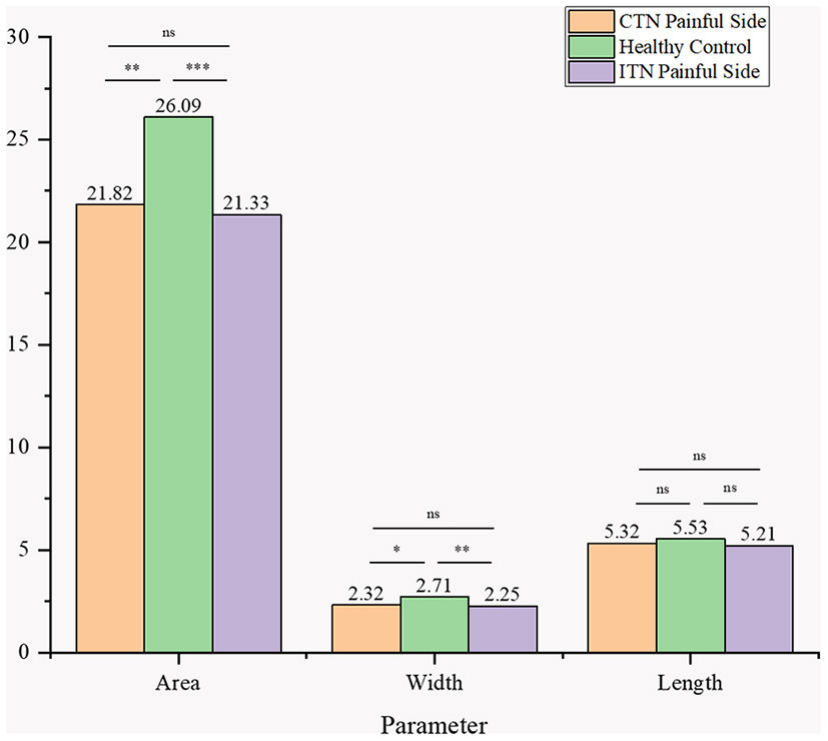
Morphometric differences in area, width, and length between the three groups were presented. ns, no significance difference; *p < 0.05, **p < 0.01, ***p < 0.001.

When compared with HCs, the area and width of ovale foramina on the painful side in ITN and CTN patients were significantly smaller. However, the difference in the width and area of ovale foramina between the ITN and CTN groups was not statistically significant. The mean length of ovale foramina was similar in the three groups and no significant difference was observed. Detailed data are present in Figure 5.

### Multivariate analysis for morphometric factors contributing to the development of PTN

Multivariate logistic regression models were constructed with factors including age, sex, area, width, and length of ovale foramina. After adjusting for covariables, it was found that smaller area and width values of ovale foramina on the symptomatic side corresponded to more likely chances that the patient would develop PTN, and this difference was statistically significant (*p* = 0.007, *p* = 0.020). As to the length, there was no statistically significant difference in the effect on PTN (*p* = 0.282). The odds ratios (ORs), 95% confidence intervals (95% CIs), and *p*-values are shown in Table 3.

**Table 3:** Odds ratios from logistic regression, adjusted for age and sex.

**Variable**	**Adjusted OR (95% CI)**	***P-*value**
Area	0.942 (0.903–0.984)	0.007^**^
Width	0.592 (0.381–0.919)	0.020^*^
Length	0.858 (0.650–1.134)	0.282

* *p* < 0.05,

** *p* < 0.01.

## Discussion

As a vital anatomical pore canal, the foramen ovale connects the middle cranial fossa and the inferior temporal fossa (35). When considering its significance in percutaneous treatments for PTN, there have been many studies (26, 36). However, most of the previous studies have only concentrated on the blocking effect on foramen ovale puncture. Currently, only a few studies have paid attention to the association between the size of ovale foramina with PTN, and there have been some defects in their methods of measurement; therefore, there is a lack of precise and reliable statistical data. Hence, several improvements have been made in the methods to ensure the accuracy of the data that we collected. We used high-resolution 3D CT and adjusted the angle to ensure that the layer that we measured was the smallest cross-section of the foramen ovale where the mandibular branch is more likely to be entrapped. In addition, stricter grouping and inclusion criteria of patients were set and more samples were included in this research. *Via* the abovementioned improvements, we deemed that the data were relatively representative and trustworthy.

In this study, we first measured and compared the morphometric characteristics of ovale foramina on the left side to those on the right side. There was no significant difference between the left and right sides in both HCs and patients with PTN. The results suggested that the higher prevalence of the right side cannot be solely explained by the differences in the size of the foramen ovale. Previous studies have demonstrated the same conclusion (26, 37–41).

Furthermore, the morphometric parameters of the ovale foramina on the painful and painless sides were measured and compared. According to the measurement results, we found that the width of the affected side was significantly smaller (*p* = 0.03) than that of the unaffected side in the ITN group. However, there was no significant difference noted in the area and length. As to the CTN group, the size of ovale foramina on the painful side was similar highly to that on the painless side, with no statistically significant difference in the morphometric characteristics observed. Then we compared the size of ovale foramina on the painful side in the ITN group to that on the painless side in the HC group, and the results suggested that the area and width of the ovale foramina on the symptomatic side of ITN patients were statistically smaller than those of HCs. Notably, when compared to the HC group, the CTN group achieved the same findings as the ITN group. The ovale foramina on the painful side were statistically narrower than the ovale foramina on the painless side of HCs. Based on the above comparison findings, the multivariate regression analysis was made and the results demonstrated that the area and width of ovale foramina were protective factors that reduced the incidence of PTN. In other words, individuals with a narrower foramen ovale are more likely to develop PTN. Taken together, these results suggested that a narrow foramen ovale may play a key role in the pathogenic mechanism of PTN. Our conclusions are partially supported by previous studies (37, 39), but there are also some observations that are contradict to those (42). For example, a study performed by Kastamoni et al. (37) demonstrated that no significant difference was found between the painful and painless sides of 19 patients with TN. Another study conducted on 21 patients provided the same conclusion. This disparity may be explained by the following points: (1) the sample size of these studies was too small to exhibit significant differences, (2) the CT images on which they measured the parameters were random (instead of being the smallest), and (3) the mandibular branch was not involved in some of the included patients.

Two different pathogenic mechanisms that may be involved in the development of PTN caused by a narrow foramen ovale are proposed in this paper and will be separately discussed in detail below. Considering the fact that the majority of TN are unilateral and no statistically significant difference in the size of bilateral ovale foramina was noted, a narrow foramen ovale may not be an independent cause of CTN, although the painful-sided ovale foramina were smaller than that of healthy individuals. However, it can be inferred that the onset of CTN may be ascribed to the trigeminal nerve compressed by blood vessels and a narrow foramen ovale meanwhile. The compression formed by a narrow foramen ovale could be secondary to NVC, which results in a swelling of the mandibular nerve, therefore making it susceptible to entrapment during its course through a narrow foramen (43–47). In recent years, it has been suggested that most individuals with NVC are asymptomatic and NVC alone may not be sufficient to cause CTN and elucidate some clinical phenomena in some CTN patients (48). Part of CTN may develop only in the case of concurrent compression of the trigeminal nerve by the vessels and the foramen ovale. The double compression may help explain some clinical observations: (1) this kind of double-crushing can show light on why some healthy individuals with NVC do not develop symptoms while others do; (2) Concerning the difference in the incidence of each branch of the trigeminal nerve, it is puzzling why CTN tends to occur in the maxillary and mandibular branches rather than the ophthalmic branch (49, 50). In our understanding, the variance may be attributed to the susceptibility of the maxillary and mandibular nerves to be compressed by narrow orifices (foramen rotundum and foramen ovale) in their pathways to the skull; (3) In a proportion of TN patients (about 28–50%) (51–53), the pain attack intervals can be followed by continuous or near-continuous background, dull pain, which is termed CTN with concomitant persistent facial pain (i.e., atypical TN) (4). This kind of atypical presentation may be due to the continuous compression by a narrow foramen ovale. In many retrospective investigations, atypical TN was found to be associated with poor outcomes after MVD (6, 54, 55). From our point of view, MVD can only relieve vascular compression of the trigeminal nerve for these atypical patients, but the compression by a narrow foramen ovale persists, which is probably the main reason for the low pain relief rate and high recurrence rate of MVD.

Besides, we propose that a narrow foramen ovale alone may be an independent and primary cause of a part of patients with ITN. Unlike CTN patients, ITN patients do not have apparent morphological changes in the REZ. Hence, we put forward another hypothesis that the chronic compression by a narrow foramen ovale leads to focal demyelination of the mandibular division, thus forming a REZ-like region in the foramen ovale. Vessels passing through the foramen ovale may be in close contact with the mandibular division in such a confined channel. In the context of the trigeminal nerve indented by a narrow foramen ovale, pulsatile compression of demyelinated axons by adjacent vessels may be the cause of abnormal impulses in ITN patients, therefore leading to the onset of throbbing pain. With the deepening recognition of NVC, the conventional perspective that compression of the trigeminal nerve is restricted to the central-peripheral myelin transitional zone has been challenged by accumulating studies (15). It has been suggested that a vessel that compresses any part of the trigeminal nerve may have secondary effects on the transitional zone, giving rise to the occurrence of pain (56, 57). On the one hand, this mechanism is supported by this research on anatomical structure and our previous results that the width of affected-sided ovale foramina was not only statistically smaller than that of HCs, but also smaller than the unaffected-sided ovale foramina of ITN patients (58). On the other hand, in terms of the ultrastructural pathological abnormalities of the trigeminal nerve, our hypothesis can still be supported by several studies. Slobodan et al. (59) pointed out that apart from the central zone of demyelination, the electron microscope examination of the trigeminal nerve also revealed alterations of the peripheral myelin and changes of the peripheral axons like atrophy or hypertrophy, neurofilaments increase and loss of the myelin occasionally. It is likely that the distal axonal demyelination is due to compression of the narrow foramen ovale.

The pathomechanisms leading to the development of TN are not completely understood until now. In recent years, an increasing number of studies have suggested that TN might be a channelopathy and that central mechanisms may be closely involved in its development (60–67). Some new drugs targeting ion channels are being developed and are already in clinical trials (68). However, the mainstream perspective is still that the trigeminal nerve is compressed at REZ by a blood vessel and the most effective treatment is MVD. In fact, the compression, which could generate an ectopic action potential from the compressed site of the axon, may arise from veins, neoplasms, or Teflon, etc. (34, 69, 70). The discovery of the association between narrow ovale foramina and the development of PTN has crucial clinical significance not only in helping understand the clinical characteristics, but also in helping neurosurgeons select appropriate surgical treatments for PTN patients. Herein, we suggest that when considering the likelihood of recurrence and poor outcomes, MVD alone may not be applicable to PTN patients with the mandibular nerve potentially being compressed by a narrow foramen ovale. Namely, if a significantly narrow foramen ovale was preoperatively noticed on the painful side on radiographic images, transcutaneous procedures instead of MVD should be given priority for neurosurgeons to achieve better clinical outcomes. In addition, for patients with recurrent PTN after MVD, it may be helpful to measure the dimension of ovale foramina before deciding on subsequent surgical treatments. Based on the abovementioned comparative analysis, we argued that a narrow foramen ovale may play a critical role in the pathogenesis of PTN, thus potentially being involved in relapse after MVD.

The research has several limitations. Firstly, it is a retrospective study in essence. The next step is to carry on prospective studies to compare the outcomes of MVD in patients with narrow ovale foramina vs. patients with normal ovale foramina. Secondly, the sample size of ITN patients is too small for the reason that ITN patients are relatively few. Thirdly, the absence of histological evidence on the trigeminal nerve at the narrow foramen ovale is a limitation of our research. Subsequent studies should focus on changes in the ultrastructure of the distal mandibular nerve axons and myelin sheaths.

## Data availability statement

The original contributions presented in the study are included in the article/supplementary material, further inquiries can be directed to the corresponding author.

## Author contributions

SL: writing—original draft, data curation, and visualization. CL: investigation, writing—review and editing, formal analysis, and validation. XY: investigation, writing—review and editing, and data collection. MQ: investigation and writing—review and editing. WZ: supervision, writing—review and editing, and resources. All authors discussed the results, commented on the manuscript, contributed to the article, and approved the submitted version.

## Funding

This work was funded by the National Natural Science Foundation of China (81771320) and Natural Science Foundation of Shanghai (21ZR1438100).

## Conflict of interest

The authors declare that the research was conducted in the absence of any commercial or financial relationships that could be construed as a potential conflict of interest. The reviewer JZ declared a shared parent affiliation with the authors to the handling editor at the time of review.

## Publisher's note

All claims expressed in this article are solely those of the authors and do not necessarily represent those of their affiliated organizations, or those of the publisher, the editors and the reviewers. Any product that may be evaluated in this article, or claim that may be made by its manufacturer, is not guaranteed or endorsed by the publisher.
